# Plant protein edible inks: Upgrading from 3D to 4D food printing

**DOI:** 10.1016/j.fochx.2025.102280

**Published:** 2025-02-13

**Authors:** Fatemeh Aghababaei, David Julian McClements, Marc Pignitter, Milad Hadidi

**Affiliations:** a Aora Health, Calle Via de los Poblados, 17, 28033 Madrid, Spain; b Department of Food Science, University of Massachusetts, Amherst, MA 01003, United States; c Institute of Physiological Chemistry, Faculty of Chemistry, University of Vienna, 1090, Austria

**Keywords:** Plant-based foods, 3D printing, Edible inks, Additive manufacturing

## Abstract

The utilization of plant proteins to formulate edible inks for 3D/4D food printing applications may help address challenges linked to food sustainability, personalized nutrition, and security. We investigate the suitability of various plant proteins for this purpose, including their molecular, functional, and nutritional attributes. Furthermore, we examine the potential of plant protein-based edible inks in 4D printing applications, where the shape or other properties of a material change over time, enabling controlled release profiles and texture modulations. We also discuss the environmental implications, regulatory considerations, and consumer acceptance of plant-based 3D/4D printed foods.

Pea and soy proteins are widely used as inks for 3D/4D food printing applications due to their excellent structure-forming abilities, as well as their functional and nutritional properties. However, solely plant protein-based inks often lack essential characteristics required for optimal performance. Their properties can be enhanced by incorporating other food ingredients, such as polysaccharides and polyphenols. As this emerging field holds promise for addressing multiple global food-related challenges, it necessitates interdisciplinary collaboration and ongoing research to unlock its full potential.

## Highlights

• Plant proteins have great potential as edible inks for 3D/4D food applications.
• Plant-based proteins can form semi-solid pastes, gels, and emulsions.
• Printing machines and parameters significantly influence the final product quality.
• Pea and soy proteins are widely used as inks for 3D/4D food printing applications.
• The most common transitions in 4D printed foods are shape and color changes.

## 1 Introduction

**Fig. 1:**
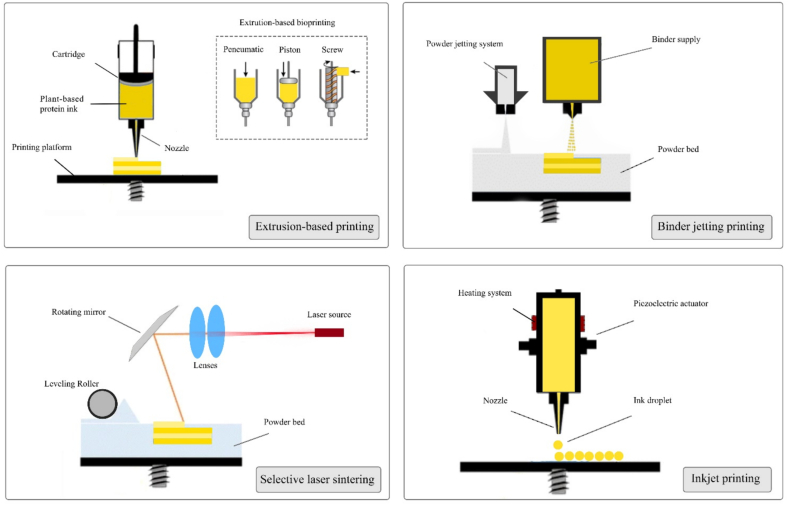
The most common design types for 3D printing technologies for food printing.

The utilization of additive manufacturing (AM) technology in the food industry has gained interest for its potential to create products with customized sensorial or nutritional attributes. The color, size, shape, texture, mouthfeel, and nutritional attributes of food products can be tailored to the specific requirements of particular consumer segments (Teng et al., 2021). 3D printing relies on computer-aided design (CAD), where digital model files are used to guide the incremental deposition of edible inks in a layer-by-layer fashion, thereby leading to the creation of 3D structures (Demei et al., 2022). In contrast to conventional shaping technologies, 3D printing allows for the creation of food materials with complex structures. 3D printing has found extensive application across diverse industries, including automotive, pharmaceutical, biomedical, textile, military, and food sectors (Chen et al., 2022). In the 3D printing for food application, a substantial body of research has focused on identifying and characterizing food materials that can be used as edible inks in 3D printing applications. In addition, researchers have focused on different kinds of additive manufacturing technologies that may be suitable for food applications, including extrusion, binder jetting, selective laser sintering, and inkjet printing (Fig. 1) (Yu, Zhang, et al., 2023).

3D printing has various potential applications within the food industry due to its ability to create foods with different morphologies, textures, and nutritional profiles. Moreover, after printing, the properties of 3D printed foods can be modified by using a variety of post-printing processing technologies, including baking, drying, frying, freezing, and sterilization (Demei et al., 2022). Recently, there has also been interest in creating 3D printed foods that change their properties (such as their size, shape, or color) after printing, which has spurred the development and application of four-dimensional (4D) food printing (He et al., 2020). The 4D printing technology is an extension of 3D printing, where an addition time axis is integrated alongside the conventional 3D coordinate axis. 4D printed products are specifically designed so that consumers experience changes in their size, shape, color, flavor, or nutrient profile in response to time or specific stimuli (Teng et al., 2021).

Given the operational capabilities of 3D/4D printing, any raw material capable of being extruded through printing nozzles and maintaining stability during molding can be transformed into the desired printing material as per the predetermined model, quality specifications, and relevant stimuli. This stands in stark contrast to the traditional methods of food processing, which heavily rely on the in-processing stage and subsequent post-processing procedures to shape the final product. This variance can be attributed to the fundamental principle of 3D/4D printing technology, which involves the deconstruction and subsequent reconstruction of food materials (Shi et al., 2024).

Moreover, the ability to successfully perform 3D/4D printing depends on the material characteristics of the edible ink used, including its printability, stability, and time-dependent dynamics. A good composite should exhibit appropriate rheological properties, viscosity, shear-thinning behavior, and rapid gelation to ensure precise deposition and structural integrity throughout the printing process. In the food industry, the major components used to formulate these edible inks are pastes, doughs, gels, and emulsions, which may be assembled from proteins, carbohydrates, lipids, and/or water. Proteins are widely used for this purpose because of their good nutritional profile and their ability to form semi-solid materials. Lately, there has been growing interest in substituting animal-derived proteins with plant-based alternatives, which is driven by the desire to improve environmental sustainability, animal welfare, and human health (Hadidi, Aghababaei, & McClements, 2023). As a result, researchers are trying to identify and characterize edible inks derived from plant-derived materials for utilization in 3D printing applications (Chen et al., 2022).

Owing to the broad scope of this field, this review delved into the understanding of how plant-based proteins can serve as environmentally sustainable protein sources in the creation of food products using 3D/4D printing technology.

## 2 Plant protein-based inks for 3D/4D printing

Consumption of sufficient quantities of protein is important for human health and wellbeing, as it provides energy and essential amino acids (Chen, Zhang, & Devahastin, 2021). Moreover, proteins exhibit a diverse range of techno-functional attributes that make them suitable as structuring materials in foods, including thickening, gelling, and emulsifying properties (Hadidi, Aghababaei, Mahfouzi, et al., 2023). Consequently, there has been great interest in utilizing proteins to create edible inks suitable for 3D/4D food printing applications. In extrusion-based 3D printing, the successful execution of printing hinges on the rheological and surface characteristics of the printing ink, which depend on its formulation (Zhu et al., 2019). Recently, there has been growing interest in using plant-derived proteins in foods instead of animal-derived ones, which is driven by the recognition that humanity must adopt a more environmentally friendly, sustainable, and ethical food supply. It is imperative to produce sufficient food to feed the growing global population without damaging the planet, which can be achieved more easily by reducing our reliance on animal-derived protein sources, like meat, seafood, egg, and dairy products (Hadidi, Hossienpour, Nooshkam, et al., 2023).

Table 1 shows a selection of plant proteins that have potential application for 3D/4D food printing. These proteins are being used to assemble plant-based foods that replicate the appearance, texture, nutritional profile, and eating experience of real meat, as well as to create novel protein-rich foods. At present, soy and pea protein isolates are the main sources of plant proteins being employed in 3D/4D food printing applications because their low cost, abundance, and functionality. It is easier to obtain pea proteins from non-genetically modified sources, which is an advantage for some applications (Goldstein & Reifen, 2022). Plant proteins can be used as edible inks because they can form pastes or gels that can be forced through a nozzle and then set (Qiu, McClements, et al., 2023). This feature is due to their ability to associate with their neighbors and form 3D networks through hydrophobic interactions, hydrogen bonding, disulfide bonds, and electrostatic interactions, which contribute to the formation of stable viscoelastic networks. Their neighbors can be other proteins (forming structured gels through intermolecular bonds), polysaccharides (*e.g.*, xanthan gum enhancing gel stability), or lipids (as in gelled oil-in-water emulsions used for edible inks). In addition, plant proteins can be used as emulsifiers to form gelled oil-in-water emulsions that are also suitable as edible inks (Feng et al., 2022). However, the functional performance of proteins is often insufficient on their own. Consequently, they need to be combined with other ingredients, especially polysaccharides, to improve their performance. For example, the printing performance of pea proteins have been improved by combining them with flaxseed gum (Liang et al., 2017). Consequently, it is important to identify appropriate combinations of ingredients to produce edible inks with the appropriate nutrient profiles and printing performance.

**Table 1:** Application of plant proteins for 3D/4D printing of foods.

**Plant protein type**	**Other material**	**Printer model**	**Printing process**	**Product picture**	**Ref**
**3D**					
Pea protein	Xanthan gum	Shiyin Tech. Co., Hangzhou, china	- Nozzle size: 1.2 mm - Printer temperature: 25 °C - Infill density: 100 %		(Liu et al., 2025)
Pea protein	Xanthan gum	A syringe extrusion printer (Shiyin Tech. Co. Ltd., Hangzhou, China)	- Nozzle size: 1.2 mm - Layer height: 1.2 mm - Printer speed: 25 mm/s - Infill density: 100 % - Printer temperature: 25 °C		(Liu, Chen, et al., 2023)
Pea protein isolate	Flaxseed gum	Extrusion 3D printer (EFL3D, not mentioned)	- Nozzle size: 0.84 mm - Layer height: 0.6 mm - Printer speed: 6 mm/s - Filling rate: 90 % - Extrusion pressure: 15 kPa		(Y. Wang et al., 2023)
Pea protein isolate	Carrageenan- and glycyrrhizic	Extrusion 3D printer (Shiyin Co. Ltd., Hangzhou, China)	- Nozzle size: 0.4 mm - Printer speed: 20 mm/s - Printer temperature: 25 °C		(Lin et al., 2024)
Soy protein isolate	Xanthan gum and rice starch	A custom-made 3D syringe pump extrusion	- Nozzle inner diameter: 200/600 μm - Extrusion flow rate: 32.56 mL/min - XY moving speed: 1200–1500 mm/min		(Shi et al., 2023)
Soy protein isolate	Guar gum and xanthan gum	Extrusion 3D printer (Shiyin Co. Ltd., Hangzhou, China)	- Nozzle size: 0.84 mm - Layer height: 0.6 mm - Printer speed: 25 mm/s - Filling rate: 90 % - Extrusion pressure: 0.36 MPa		(Yu, Li, et al., 2023)
Faba bean protein isolate	–	Wiiboox Sweetin chocolate syringe extrusion type printer (Wiiboox, China)	- Nozzle size: 0.84 mm - Printer speed: 25 mm/s - Layer height: 0.5 mm - Printer temperature: 20 °C		(Hu et al., 2023)
Zein	Soybean oil, glycyrrhizic acid	Extrusion 3D printer (Shiyin Co. Ltd., Hangzhou, China)	- Nozzle size: 0.40 mm - Printer speed: 15 mm/s - Printer temperature: 25 °C		(Qiu, Wang, et al., 2023)
Zein	Tannic acid, sodium alginate	3D printer (FOODBOT-S2, China)	- Nozzle inner diameter: 1.2 mm - Nozzle height: 2.5 mm - Nozzle movement speed: 20 mm3/s and 30 mm^3^/s - Infill density: 100 % - Extrusion speed:10 mm^3^/s		(Liu, Xie, et al., 2023)
Soy protein isolate	Rice protein, and wheat gluten	Extrusion-based 3D printer (Nanchang University, Bioprinter-I)	- Nozzle size: 0.84 mm - Layer height: 0.84 mm - Retraction speed: 8 mm/s - Printer speed: 20 mm/s - Filling density: 100 % - Printer temperature: 25 °C		(Qiu, McClements, et al., 2023)
Spirulina Platensis protein	Soybean oils, sunflower waxes, and xanthan gums	Food 3D printer (E1, Porimy, China)	- Printing rate: 20 mm/s - Printer speed: 25 mm/s - Printer temperature: 25 °C		(Guo, Gu, et al., 2023)
Soy protein isolate	Guar gum and xanthan gum	Extrusion 3D printer (Shiyin Co. Ltd., Hangzhou, China)	- Nozzle size: 0.84 mm - Layer height: 0.6 mm -Printer speed: 25 mm/s - Infill density: 90 %		(Yu et al., 2022)
Pea protein isolate	Pullulan mixture	Extrusion 3D food printer (YOLILO Co., Ltd.)	- Nozzle size: 1.1 mm - Layer height: 0.9 mm -Printer speed: 20 mm/s		(Lim et al., 2022)
Soy protein isolate	Flammulina velutipes polysaccharide	3D printer (FoodBot-D1, Shinnove, Hangzhou, China)	- Nozzle size: 1.2 mm - Layer height: 1 mm -Filling rate: 80 % - Nozzle movement rate: 15 mm/s - Printer temperature: 25 °C		(Pan et al., 2022)
Gluten	Tween-80, starch, cocoa butter, sodium alginate, and shiitake mushroom powder	3D printer (Willboox Technology Co., Ltd.)	Nozzle diameter (0.84 mm)		(S. Wang & Liu, 2022)
Pea protein isolate	High methoxyl pectin, epigallocatechin gallate, and cinnamaldehyde	Extrusion 3D printer (Shiyin Co. Ltd., Hangzhou, China)	- Nozzle size: 0.84 mm - Layer height: 0.75 mm - Retraction speed: 50 mm/s - Printer speed: 35 mm/s - Filling density: 10 % - Printer temperature: 25 °C		(Feng et al., 2022)
Peanut protein isolate	Vegetable and fruit powder, sodium alginate	Extrusion printer (FOODBOT-MF, China Changxing Shiyin Technology Co., Ltd.)	- Syringe diameter: 22 mm - Nozzle size: 0.8 mm - Printer speed:1200 mm/min		(Chen, Zhang, & Phuhongsung, 2021)
Soy protein isolate	Sodium alginate and gelatin	Extrusion-based 3D printer (3.0, Felix, The Netherlands)	- Nozzle size: 1.55 mm - Volume of syringe: 60 mL - Layer height: 0.66 mm - Shell thickness: 1.5 mm - Printer speed: 10 mm/s - Flow rate: 80 % - Printer temperature: 35 °C		(Chen et al., 2019)
Pleurotus ostreatus protein	κ-carrageenan	Extrusion-based double-syringe printer (Shiyin Tech. Co. Ltd., Hangzhou, China)	- Nozzle size: 1 mm - Printer speed: 25 mm/s - Printer temperature: 25 °C		(Z. Liu et al., 2022)
Soy protein isolate	Strawberry powder	Extrusion 3D printer (Shiyin Co. Ltd., Hangzhou, China)	- Nozzle size: 1.22 mm - Nozzle moving speed: 17 mm/s - First layer height: 0.9 mm		(Fan et al., 2020)

**4D**					
Pea protein isolate	Purple sweet potato flour	Ultimaker^2+^ (Ultimaker B.V., New York, USA)	- Nozzle size: 0.4–0.8 mm - Filling degree: 100–108 % - Printer speed: 15 mm/s - Printer temperature: room temperature - 4th dimension: pH-dependent color change of anthocyanin		(Su et al., 2025)
Pea protein isolate	Xanthan gum	Shinnove, Hangzhou Shiyin Technology, Hangzhou, China)	- Nozzle size: 1.1 mm - Printing rate: 25 mm/s - Flow rate: 100 % - Infill density: 90 % - Printing time: 10.65 min - Printer temperature: 30 °C - 4th dimension: shape change by hot air drying		(Koirala et al., 2025)
Peanut protein isolate	Carrageenan and gellan gum	Extrusion 3D printer (Shiyin Co. Ltd., Hangzhou, China)	- Nozzle size: 0.4 mm - Printing rate: 12, 20, 30 mm/s - Filling degree: 10 % - Printer temperature: 65 °C - 4th dimension: pH-dependent color change of anthocyanin		(Lin et al., 2023)
Soy protein isolate	k-carrageenan and vanilla flavor	Extrusion 3D printer (Shiyin Co. Ltd., Hangzhou, China)	- Nozzle size: 1.2 mm - Nozzle moving speed: 20 mm/s - First layer height: 1.2 mm - Extrusion rate: 0.044 mm/s - Infill density: 60 % - 4th dimension: change color and flavor by microwave heating		(Phuhongsung et al., 2020)
Soy protein isolate	Oat flour, carmine and glycerin	FoodBot MF 3D	- Nozzle size: 0.8 mm - Layer height: 0.8 mm - X/Y axis speed: 17 mm/s - Z axis speed: 16.7 mm/s - Filling rate: 90 % - Printer temperature: 25 °C -4th dimension: shape change by microwave heating		(Guo, Zhang, et al., 2023)

Several researchers have examined the formulation of edible inks from plant proteins. For instance, the rheological properties and printability of a blend comprised of soy protein isolate, sodium alginate, and gelatin has been examined (Chen et al., 2019). It's worth noting that gelatin, being an animal-derived protein, is not suitable for use in plant-based foods. Other researchers have demonstrated the printability of edible inks based on broad bean protein, milk powder, starch, and their combinations (Lille et al., 2018). In another study, researchers investigated the impact of pea protein on the 3D printing characteristics of potato starch. Nevertheless, the range of proteins that have been explored for their potential application in 3D printing remains relatively restricted.

Recently, researchers characterized the rheological characteristics and printing capabilities of edible inks formulated from rice protein (RP), soy protein isolate (SPI), and wheat gluten (WG) (Qiu, McClements, et al., 2023). The objective was to blend SPI, WG, and RP powders to formulate a high-protein edible ink, constituting of 25 % of dry matter content, which could then be utilized for the fabrication of 3D-printed meat analogs. The viscosity, shear modulus, and printability of these blended protein pastes could be manipulated by altering their composition. Indeed, the protein paste composition could be adjusted to generate printed objects with customizable structures, textures, printing precision, and printing stability, making them suitable for developing 3D-printed meat substitutes (Qiu, McClements, et al., 2023).

In another recent study, Lin et al. (2023) developed semi-interpenetrating networks comprised of peanut protein and polysaccharides (carrageenan and gellan gum) to enhance the printing and setting characteristics of composite hydrogels. The incorporation of the polysaccharides notably improved the mechanical attributes of the hydrogels. The edible inks created exhibited thermo-reversible cold-set characteristics due to the presence of the polysaccharides. Thus, they could be extruded through the nozzle at high temperatures and then set by depositing them on a cold platform.

## 3 Shift from 3D to 4D printing

**Fig. 2:**
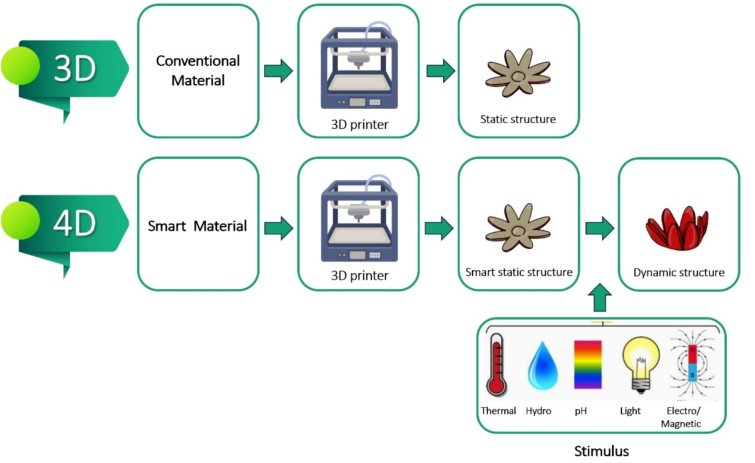
Summary of the differences between 3D and 4D food printing techniques.

4D printed objects are intelligent materials whose properties may change in response to alterations in environmental conditions or specific triggers, such as time, pH, ionic strength, temperature, light, or water content (Ramesh et al., 2018). Edible inks suitable for 4D food printing applications are usually created by blending food ingredients that can change in response to environmental factors. The differences between 3D and 4D printed foods are depicted in Fig. 2. Some potential advantages of 4D printing are highlighted below:

(a) *Novel eating experiences*. Foods that provide novel eating experiences can be produced by creating edible objects that undergo transitions in their properties after printing. For instance, edible flowers that bloom on your plate can be created. The transformation of the flowers from a closed state to full bloom adds an intriguing dimension to the dining experience. Thus, 4D printed food allows for innovative food design, increasing the engagement between diners and foods (Ramesh et al., 2018).
(b) *Controlled release*: Another potential benefit of 4D printing is the ability to control the release of flavors, colors, or nutrients in the printed product while the user consumes it (Ghazal et al., 2023). Consequently, desirable sensorial experiences or increased nutrient efficacy can be achieved.
(c) *Flat packaging:* Edible objects can be printed into 2D structures (“flat packaging”) that expand into 3D structures after printing (Teng et al., 2021). This may be useful for reducing transport and storage costs.

### 3.1 Appearance

There are several factors that should be considered when creating 4D printed foods. First, it is important that the materials used to formulate the edible inks can be extruded through a nozzle and set after printing. Second, it is crucial to utilize materials that can respond to environmental triggers, such as time, temperature, moisture content, pH, or light. Third, the transition kinetics from one state to another is critical, *e.g.,* does the transition take seconds, minutes, or hours (Ghazal et al., 2019). Currently, the most common transitions used in 4D printed foods involve changes in shape or color.

The investigation into color changes induced by stimuli is a focal point in research on 4D-printed food. These shifts in color have transformed into optical features that buyers utilize to evaluate the product's value prior to purchasing. Additionally, color can impact customers' perception of the potential flavor and taste of the product (Singh et al., 2024). As a result of climate fluctuations, certain colors may alter, and changes in pH levels can modify the chemical structure. For instance, some anthocyanin and curcumin displayed green and red hues in alkaline pH conditions, but under acidic conditions, they shifted to red and yellow, respectively. This capability can be harnessed in 4D-printed food to alter various shades over time by manipulating stimuli such as pH, temperature, and other factors. For instance, researchers utilized curcumin as a stimulus to examine the impact of microwave stimulation on lotus root powder gel during 4D printing (Chen, Zhang, Guo, & Chen, 2021). As a stimulus-response material, a mixture of compounds comprising gelatin-gum and Arabic-oil complexed with accrued microparticles was employed. Microwave treatment led to an enhancement in the reddish color of the dough while concurrently reducing its lightness. This phenomenon occurs because the capsule is disrupted by microwave exposure, resulting in the release of encapsulated oil. The color change of the soy protein, beetroot, and pumpkin combination, rich in betalain, was investigated in relation to surface pH in isolated 3D-printed structures. The researchers discovered that after 1 h of preservation at higher pH levels (above 6), the color intensity of the printed material remained largely unchanged. However, significant alterations in color values were observed at lower pH levels (Phuhongsung et al., 2020). Color values fluctuate over time due to the degradation of color pigments caused by oxidation. The changes in color value at lower pH levels stem from the degradation of color pigments in highly acidic conditions. A significant color change was observed in the multi smart material structure used in 3D printing in response to both internal and external pH stimuli (Ghazal et al., 2023).

Food hydrogels are utilized in food printing due to their ability to asymmetrically swell and change shape. Hydration-induced shape modification facilitates programmable metamorphosis, providing a unique mouthfeel and enabling convenient shipment and storage. Cooking induces an asymmetrical swelling process that transitions the dimensions of a composite material consisting of cellulose, protein, and starch from 2D to 3D. Hydrogels possess a three-dimensional network structure formed through either chemical or physical interactions. Comprising expanding hydrophilic substances, they facilitate shape-shifting in response to diverse signals (Singh et al., 2024). 4D food printing has the potential to revolutionize the manufacturing of texturized protein and meat. Traditional meat production is known for its resource-intensive and environmentally damaging practices. By utilizing plant-based proteins and other ingredients, 4D food printing offers a more sustainable approach to creating meat-like products. In 4D food printing, layers of food are meticulously deposited onto a platform in a precise pattern, allowing for the creation of intricate structures that closely resemble meat. Additionally, flavorings, minerals, and bioactive chemicals can be incorporated into printed products to enhance their nutritional content and flavor (Yang et al., 2021). Drying, a fundamental food processing technique, can lead to changes in the shape and texture of food items. During dehydration, water is removed from the sample, resulting in stress on the food and causing product shrinkage and bending. The folding angle of a 3D-printed item exhibits a positive correlation with moisture content and shrinking ratio, while demonstrating a negative correlation with the dielectric constant loss factor (C. Chen et al., 2021).

### 3.2 Flavor and nutritional content

Food printing technology plays a crucial role in creating unique flavors by combining various components to promote favorable reactions. *E*-tongue analysis assesses the changes in taste qualities of printed food items, utilizing eight distinct sensations that mimic human tongue experiences, including sourness, bitterness, sweetness, aftertaste, umami, creaminess, and saltiness (Ghazal, Zhang, Bhandari and Chen, 2021, Ghazal, Zhang, Mujumdar and Ghamry, 2023). A GC–MS analysis and electronic nose can be used to examine fluctuations in the constituents of the flavor of 3D-printed food structures. Treatment of samples at higher pH levels (pH 9 and 10) had a reduced response, whereas samples treated at lower pH levels exhibited a greater reaction. Vanillin, a phenolic compound with a pKa of 7.38, exhibits reduced volatility in basic conditions and may become negatively charged, leading to diminished odor intensity at higher pH levels. pH-induced alterations in flavor attributes have also been observed in the multi-smart components of 4D-printed food. Across different pH levels (2−10), a decrease in pH led to an increase in sourness, while saltiness and umami flavors decreased (Ghazal et al., 2021). Similarly, in a 3D-printed mixture of soy protein extract, beetroot, and pumpkin, a high salty flavor of 10 was observed, with stronger astringency noted at a lower pH of 4. The pH of the sprayed solution decreases as the number of hydrogen ions increases at lower pH values (Phuhongsung et al., 2020). The interaction of sodium protein leads to the release of sodium from printed objects, resulting in increased saltiness at high pH levels. Vanillin, a food flavoring agent, exhibits flavor intensity that is highly sensitive to various stimuli such as pH, temperature, and moisture (Singh et al., 2024). The study examined how microwave irradiation impacted 4D printed objects that utilized vanilla flavoring in the printing ink. Researchers extensively employed vanillin as a stimulus-responsive material in the 4D printing method (Ghazal et al., 2021; Phuhongsung et al., 2020).

Apart from flavor, food printing offers the potential to customize the nutritional content of food according to customer preferences. This can be achieved by incorporating health-promoting elements such as cellulose, plant compounds, and modified proteins, while minimizing the presence of harmful chemicals such as allergens and anti-nutritional compounds. The nutritional composition of printed food can be controlled through strategic placement of nutrients in inks or through alternative printing techniques. Fruits and vegetables can contribute vitamins, polysaccharides, and various other ingredients to food ink (Y. Chen et al., 2022). Tissue engineering holds potential in 4D printing to improve the nutritional characteristics of food. By subjecting the material to optimal conditions, tissue growth can be stimulated. Printed objects can consist of animal or plant cells that, when activated, have the capability to form tissue-like structures and generate nutrients (Teng et al., 2021). Incorporating microflora into printed food enhances the nutritional quality of the final product. The inclusion of microalgae introduces functional and nutritional components like protein, sterols, vitamins, and fatty acids (Singh et al., 2024). UV irradiation significantly elevated the vitamin D2 content in 4D-printed purple sweet potato pastes supplemented with ergosterol, resulting in enhanced vitamin D2 levels in the irradiated area. Similarly, microwave-stimulated oleo gel powder ink of purple potato was utilized to produce a reduced-fat printed product based on beeswax (J. Chen et al., 2021).

The choice of food materials, printing process, and post-processing steps can influence the nutritional content of food during 4D food printing. This encompasses the selection of food materials, the printing process, and post-processing stages. The nutritional value of printed food is determined by the food materials utilized in the printing ink. Whole grains, fruits, and vegetables contribute fiber, vitamins, and minerals to printed dishes, in contrast to processed ingredients. The printing process can also impact food nutrition. Factors such as printing temperature, speed, and exposure to light can affect nutrient retention. High temperatures during printing can degrade heat-sensitive vitamins, while extended light exposure may reduce the vitamin C content in certain foods. Post-processing activities like drying, heating, and freezing can further influence the nutritional quality of printed food. While drying may concentrate nutrients, it could also lead to the destruction of heat- and oxidation-sensitive vitamins (C. Chen et al., 2021; J. Chen et al., 2021; Shi et al., 2024). Cooking has the potential to degrade heat-sensitive vitamins, although it can also enhance nutrient digestion. 4D food printing enables customization and enhances dining experiences (He et al., 2020). Thorough assessment of potential nutritional changes during printing is vital to ensure that printed food retains its nutritional value and supports a balanced diet. Choosing nutrient-rich materials, optimizing printing conditions, employing protective packaging, and minimizing post-processing can help preserve the nutritional integrity of 4D-printed food.

## 4 Characteristics of final product quality

Apart from providing essential energy, foods crafted through 3D/4D printing offer diverse qualities akin to conventional reconstituted foods. These include distinctive swallowing sensations derived from specific rheological properties and nuanced taste variances resulting from unique texture attributes. The attributes of these final products are closely linked to the printing technology utilized. The majority of 3D printing methods employed rely on extrusion printing technology, particularly Fused Deposition Modeling (FDM). In FDM for food 3D printing, the interplay between particle size, nozzle diameter, flow rate, and pressure significantly impacts printability and the stability of the printed shape. The particle size must be much smaller than the nozzle diameter to ensure smooth extrusion and prevent clogging, as larger particles can cause irregular flow and disrupt layer formation. The nozzle diameter influences the flow rate, with larger nozzles allowing faster printing but reducing precision, while smaller nozzles enhance detail resolution but require carefully controlled flow rates to maintain consistency. The flow rate is directly affected by the pressure exerted during extrusion; higher pressure facilitates faster flow but risks over-extrusion, leading to distorted structures, whereas insufficient pressure can cause weak, incomplete layers. Additionally, the particle size of the slurry plays a crucial role in the stability of the printed shape, as smaller, uniform particles promote better dispersion and network formation, leading to stronger, more stable structures. Conversely, larger or uneven particles can result in phase separation, weakening the structural integrity and causing collapse or deformation post-printing. Maintaining an optimal balance between these parameters is essential for smooth extrusion, structural integrity, shape stability, and consistent texture, ultimately enhancing the quality of the final printed product.

Research focusing on the quality of final products primarily emphasizes the characteristics of extruded colloidal/emulsion foods. Parameters directly linked to the capability and quality of samples, such as rheological analysis, texture analysis, printability, and stability, have emerged as the primary research metrics (Shi et al., 2024). However, to our current understanding, there is a lack of information regarding the performance of plant protein ingredients and how the protein content impacts the printability of plant-based pastes. Additionally, while rheological properties can be easily measured using instrumental methods like rheometers, there is a lack of well-defined definitions and quantifications for printability. Moreover, there are no standardized methods available, making it difficult for researchers to establish quantitative correlations between rheology and printability.

### 4.1 Rheological properties

3D printing technology imposes stringent criteria on the rheological properties of the food materials used for printing, referred to hereafter as “food inks”. Hence, to employ plant proteins as food inks effectively, it's crucial to enhance our comprehension of their rheological properties. This understanding is vital for predicting their printing performance in extrusion-based printing processes. Adequate mechanical strength is essential for supporting the structure of 3D-printed objects. Materials with a higher storage modulus (G') have demonstrated greater capacity for retaining shape in extruded objects (Z. Liu et al., 2018). Nevertheless, solely possessing a high G' is insufficient to determine the quality of ink printability. It's also essential to have a certain level of resistance to external stresses, such as yield stress.

In a study conducted by Ainis et al. (2023), investigated the viscoelastic properties of pea and soy protein pastes with concentrations ranging from 10 to 21 %*w*/w, and their correlation with “printability” after extrusion 3D printing. According to the results, changes in protein concentration had an impact on rheological parameters such as G´, tanδ, and σy, leading to distinct viscoelastic behaviors in pastes made from pea protein isolate and soy protein isolate. At protein concentrations ranging from 10 % to 16 % (*w*/w), SPI exhibits greater elasticity compared to pea protein isolate. However, when protein concentrations exceed 17 % (w/w), the rheological behavior of both pea protein isolate and soy protein isolate becomes similar. Within the protein concentration range of 15 % to 17 % (w/w), soy protein isolate demonstrated superior stability in forming 3D printed objects when compared to pea protein isolate. This superiority stems from soy protein isolate's inherently elastic structure, which enhances stability and mitigates the risk of collapse during the 3D printing process. As protein concentrations for pea protein isolate increased further, the rise in G´, σy, and K offset the significance of n and tanδ, leading to the creation of self-supporting 3D printed products comparable to those formed with soy protein isolate (Ainis et al., 2023). This indicates that as G' (storage modulus), σy (yield stress), and K (consistency index) increased, the material exhibited greater elasticity, structural integrity, and resistance to flow, which are essential for maintaining the shape of 3D-printed products. Meanwhile, the reduced significance of n (flow behavior index) and tan δ (loss tangent) suggests a transition towards more solid-like behavior, ensuring improved printability and self-supporting structures similar to those formed with soy protein isolate.

### 4.2 Printability

‘Printability’ refers to the capacity of a food ink to smoothly extrude from the nozzle, shaping a predetermined form capable of bearing the weight of the product without substantial alterations post-extrusion in 3D printing. Recently, a quantitative approach to defining ‘printability’ involves comparing the dimensions of the printed object with the initially designed specifications (Ainis et al., 2023). Evaluation of 3D/4D printing foods involves assessing printing performance, stability, and accuracy. These factors directly influence the quality and outcome of printed objects. Various metrics and methods are employed to gauge the effectiveness of printing processes and materials. One crucial aspect of evaluating printing quality is precision. Printing precision is essential for ensuring the repeatability of 3D printing and the fidelity of the final product (Shi et al., 2024). Achieving optimal precision relies heavily on the viscosity of the printing inks. Inks with appropriate viscosity levels facilitate smooth extrusion and enhance the overall performance of the final product. Higher precision leads to more consistent printing results and fewer defects in the final products (Ji et al., 2022).

To facilitate the evaluation process, simple geometric shapes like cubes, cylinders, and cones are often printed as test objects. These shapes allow for easy measurement and calculation of printability metrics (Ghazal et al., 2023). Additionally, textual descriptions of printing performance are commonly provided in research papers, particularly in the results and discussion sections. These descriptions often highlight the effectiveness of ink compositions in facilitating smooth extrusion and the stability of printed structures. With the growing demand for plant protein-rich foods, there's a greater need for understanding the printability of individual plant protein-based ingredients. This understanding is essential for developing multicomponent products like meat analogues.

Venkatachalam et al. (2023) formulated a diverse range of printable pea-based compositions by adjusting macronutrient composition through water content optimization. The developed formulations' printability was assessed based on extrudability (measured by extrusion force), buildability (evaluated through flow point), and printing precision (quantified by surface defect index). The extrudability of pea-based formulations was significantly impacted by their macronutrient composition, showing a strong correlation with buildability. Microscopic analysis utilizing controlled low-strength material (CLSM) revealed that the prerequisite for good printability was the formation of a continuous pea protein phase with dispersed insoluble pea fiber fibrils. Maintaining consistent printing settings, canny edge detection facilitated the measurement of printing precision. The printing precision of pea-based formulations exhibited variation depending on their macronutrient composition.

### 4.3 Microstructure

3D/4D food printing entails the breakdown and rebuilding of food components. Consequently, the resulting food is essentially structured based on the shape and formula specified by the operator on a macroscopic level. The most immediate sensory assessment of food consumption is derived from analyzing the rheology, texture, and printing effect quality of the printed products. At the micro level, the effects of 3D printing processing on food components often necessitate examining the microstructure of printing raw materials or finished products. This observation helps elucidate the interaction between internal components of the product and provides a fundamental explanation for the resulting quality outcomes. Various analytical techniques including scanning electron microscopy (SEM), fourier-transform infrared spectroscopy (FTIR), X-ray diffraction (XRD), small-angle X-ray scattering (SAXS), controlled low-strength material (CLSM), low-field nuclear magnetic resonance (LF-NMR), transmission electron microscopy (TEM), atomic force microscopy (AFM), and optical microscopy have been employed to examine the microphysical structure, molecular structure, crystal structure, nano-aggregated structure, component distribution, water distribution, submicroscopic structure, and surface morphology of super reconstructed foods. Additionally, these techniques have been utilized to assess the visual appearance of the printed products. SEM stands out as the most frequently employed method for observing microstructure due to its features including large depth-of-field, ultra-high resolution, intuitive imaging, three-dimensional intensity, wide magnification range, and the capability of rotating and tilting samples in 3D space (Relucenti et al., 2021). SEM is commonly utilized to assess various aspects of the surface structure of printed food, including compactness, presence of voids, smoothness, clarity of fiber structure, morphological integrity, and aggregate size. It serves to evaluate experimental variables such as additive dosage, printing parameter settings, and the impact of pre- and post-processing on the structure of printed products. LF-NMR has gained increased popularity for detecting water distribution in foods, particularly in 3D/4D printing applications involving high-moisture inks like hydrogel, surimi, and fresh food (Shi et al., 2024).

## 5 Challenges in 3D/4D food printing

There are several challenges that need to be addressed before 3D/4D food printing applications become more widespread, which are related to the printing technology and the formulation of edible inks with the required properties. Ensuring good printing precision and stable printed shapes are important for 3D food printing applications. Achieving precise printing necessitates an understanding of how the characteristics of food materials influence their printability (Fan et al., 2020). For 3D printing, it is critical to formulate edible inks that exhibit the required functional attributes, such as the ability to flow through a nozzle and then set after printing. This usually requires the utilization of plastic materials that have a yield stress, below which they act as solids but above which they act as fluids. Pastes, gels, and emulsions prepared from food proteins, polysaccharides, and/or lipids can be used to create this kind of edible ink. In some cases, edible inks may be fluids that flow through the nozzle and then are gelled afterwards, usually in response to an environmental trigger, such as heating, cooling, or gelling agents. At present, there is still a relatively poor understanding of how to formulate edible inks from different kinds of food ingredients. This is especially true for 4D printing applications where the edible ink should also have the ability to change its properties after printing. Teng and co-workers (Teng et al., 2021) highlighted the importance of developing structure-function relationships to create edible inks with improved characteristics. A summary of some of the main challenges encountered when employing 3D/4D food printing is given in Table 2.

**Table 2:** Advantages and disadvantages of 3D and 4D printing in food.

**Aspect**	**Advantages of 3D Printing**	**Disadvantages of 3D printing**	**Advantages of 4D Printing**	**Disadvantages of 4D printing**
Customization	- Personalized nutrition and diets - Tailored shapes, textures, and flavors - Addressing dietary restrictions/allergies - Novel and artistic food designs	- Complexity in creating customized recipes - Limited choice for certain dietary needs - Requires customer input and data	- Enhanced customization with dynamic changes - Adaptation to environmental factors - Novel and artistic food designs - Customer interaction with food over time	- Complex programming for shape and texture - Limited use cases - Complexity in material design - Technical challenges in scaling up
Production efficiency	- Efficient and precise ingredient usage - Reduced food waste - On-demand and decentralized production - Potential for automated mass production	- Initial high equipment and material costs - Slower production speed compared to traditional methods	- Reduced food waste - On-demand and decentralized production - Potential for automated mass production - Efficiency in processing and self-assembly	- Slower production speed compared to traditional methods - Technical challenges in material transformation
Food Safety	- Improved food safety through controlled processing - Reduced risk of contamination and spoilage - Enhanced traceability and transparency	- Challenges in maintaining hygiene and sanitation - Regulatory and quality control hurdles - Limited shelf life for some printed foods	- Improved food safety through controlled processing - Reduced risk of contamination and spoilage - Enhanced traceability and transparency	- Regulatory and quality control hurdles - Limited shelf life for some printed foods
Sustainability	- Reduces packaging waste - Lower transportation and storage costs - Promotes use of local and seasonal ingredients - Sustainable protein sources like algae or insects	- Energy consumption in printing process - Environmental impact of materials used - Recycling and disposal challenges - Limited availability of 3D food printers	- Reduces packaging waste - Lower transportation and storage costs - Promotes use of local and seasonal ingredients - Sustainable protein sources like algae or insects	- Environmental impact of materials used - Recycling and disposal challenges
Innovation	- Encourages culinary creativity - Experimentation with new textures and structures - Encourages collaboration between food and tech industries - Potential for space food and disaster relief applications	- Requires skilled operators and chefs - Intellectual property concerns	- Encourages culinary creativity - Experimentation with new textures and structures - Encourages collaboration between food and tech industries - Potential for space food and disaster relief applications	- Intellectual property concerns

Some characteristics of plant proteins currently limit their utilization for creating edible inks for 3D/4D food printing. Some plant proteins can be used to form gels, pastes, or emulsions under appropriate conditions (such as concentration, moisture content, temperature, pH, and ionic strength), but these conditions still need to be elucidated for different plant protein types. In some cases, plasticizers are required to create edible inks with the required mechanical properties, *e.g.,* to reduce brittleness and increase plasticity. However, excessive use of plasticizers may compromise the mechanical, sensory, and nutritional properties of edible inks. Thus, the precise type and quantity of plasticizer required needs to be established for specific applications. Emulsion technology has great potential for improving the functional performance of edible inks, but again, more research is required to optimize the concentration and size of the plant protein-coated oil droplets in these materials (Rocca-Smith et al., 2016).

The efficacy of 3D/4D food printing can also be improved by combining it with other technologies. For instance, microwave and ultrasonic technologies can be used for pre-printing and post-printing treatments to enhance the accuracy of printing and the stability of printed shapes (Xu et al., 2020).

## 6 Conclusion and future prospectives

In conclusion, the integration of plant protein-based 3D/4D printing technology in the food industry represents a promising avenue for addressing numerous challenges, including sustainability, personalized nutrition, and food security. Plant proteins hold significant potential for formulating edible inks for 3D/4D food applications due to their ability to form semi-solid pastes, gels, and emulsions. Among them, proteins from peas, soybeans, and peanuts exhibit unique functional characteristics, allowing for the development of edible inks with customizable properties. However, previous studies have shown that edible inks formulated solely from plant proteins rarely exhibit the necessary functional properties. Therefore, researchers have been focusing on enhancing protein performance in edible inks by combining them with other food ingredients, such as polysaccharides and polyphenols. This article has reviewed different plant protein sources, including their functional and nutritional properties. 4D food printing introduces dynamic responsiveness in printed food products, thereby allowing for customized flavor release and texture changes before or during consumption. This novel dimension enhances the sensory experience of food and opens up new possibilities for culinary innovation.

Despite the immense potential of plant protein-based 3D/4D printing, there are still challenges to overcome. These include improving printing precision, optimizing printing materials, and advancing our understanding of the interaction between material properties and sensory attributes. Furthermore, further research is required to formulate edible inks with predictable and innovative printing properties. Future research in this field should focus on interdisciplinary collaboration, incorporating smart materials, and developing comprehensive models for material-process-performance-function relationships. Additionally, research efforts should continue to explore the practical application of 3D/4D printed food, addressing consumer acceptance, regulatory considerations, and environmental implications.

## CRediT authorship contribution statement

**Fatemeh Aghababaei:** Writing – review & editing, Writing – original draft, Software, Methodology, Investigation, Data curation. **David Julian McClements:** Writing – review & editing, Writing – original draft, Visualization, Validation, Supervision. **Marc Pignitter:** Writing – review & editing, Writing – original draft, Visualization, Validation, Supervision. **Milad Hadidi:** Writing – review & editing, Writing – original draft, Visualization, Validation, Supervision, Methodology, Investigation, Conceptualization.

## Declaration of competing interest

The authors declare that they have no known competing financial interests or personal relationships that could have appeared to influence the work reported in this paper.

## Data Availability

No data was used for the research described in the article.
